# Wolf-Pack-Inspired Distributed Encirclement for UAV–USV Systems via Visual Connectivity Preservation

**DOI:** 10.3390/biomimetics11070513

**Published:** 2026-07-21

**Authors:** Jinheng Xiao, Shihan Kong, Jinan Sun, Yingnan Li, Fang Wu, Junzhi Yu

**Affiliations:** 1 School of Aerospace Engineering, Beijing Institute of Technology, Beijing 100081, China; 1120220683@bit.edu.cn; 2 The State Key Laboratory for Turbulence and Complex Systems, School of Advanced Manufacturing and Robotics, Peking University, Beijing 100871, China; 3 National Engineering Research Center for Software Engineering, Peking University, Beijing 100871, China; sjn@pku.edu.cn; 4 SPIC Nuclear Energy Co., Ltd., Yantai 100088, China; liyingnan@spic.com.cn (Y.L.); wufang@spic.com.cn (F.W.)

**Keywords:** heterogeneous UAV–USV systems, distributed encirclement, communication-constrained environments, biological coordination, visual connectivity preservation, wolf-pack predation

## Abstract

This paper presents a wolf-pack-inspired distributed encirclement framework for heterogeneous unmanned aerial vehicle–unmanned surface vehicle (UAV–USV) teams operating with limited communication and intermittent visual sensing. The proposed growth-based visual connectivity encirclement (GB-VCE) method separates high-level role evolution from low-level motion execution. Each agent accumulates local evidence from target observation, visual connectivity contribution, motion stability, and platform characteristics, enabling smooth transitions among leader, relay, searcher, and encircler roles. Visual links and relay relations are treated as coordination resources rather than auxiliary sensing constraints, allowing target-related information to propagate through the team without centralized fusion or persistent all-to-all communication. Simulations with paired random seeds show that GB-VCE improves encirclement accuracy, target-information coverage, convergence consistency, and heterogeneous role complementarity compared with fixed-role, static-leader, UAV-only, and USV-only baselines. The results indicate that biomimetic role growth and visual connectivity preservation provide an interpretable and robust coordination principle for air–sea cooperative encirclement.

## 1. Introduction

Encirclement is a demanding cooperative behavior in which multiple agents must maintain target observability, safe spacing, angular distribution, and robustness to target motion. The problem is particularly challenging for heterogeneous UAV–USV teams: UAVs provide fast repositioning and wide-area visual relay, whereas USVs offer persistent surface-level monitoring but slower dynamics. In maritime missions, continuous communication and global state fusion are often unavailable because of occlusion, bandwidth limits, platform motion, and environmental disturbance. Thus, the central question is how to generate distributed encirclement from local perception while preserving the flow of target-related information.

Table 1 lists the comparison of representative methods for cooperative encirclement and heterogeneous coordination. Existing approaches include geometric enclosing control, optimization and learning-based coordination, and connectivity-aware multi-robot control. Chen et al. [1] developed a monocular vision-based target-enclosing method for USVs under field-of-view constraints and uncertain dynamics. Chen et al. [2] further investigated bearing-only cooperative enclosing for multiple USVs with unknown dynamics and sideslip. Mnih et al. [3] established a deep reinforcement learning framework, while Van Hasselt et al. [4] improved value-estimation stability through double Q-learning. Orr and Dutta [5] reviewed the application of multi-agent deep reinforcement learning to multi-robot systems. Busoniu et al. [6] summarized the principles and challenges of multi-agent reinforcement learning. Lowe et al. [7] proposed a multi-agent actor–critic framework for mixed cooperative–competitive environments. Rashid et al. [8] introduced monotonic value-function factorization for cooperative multi-agent learning, whereas Foerster et al. [9] developed counterfactual policy gradients for multi-agent credit assignment. Although these learning-based approaches improve adaptability, their functional roles are generally implicit. Müller and Hutter [10] studied cooperative vision-based navigation for heterogeneous robot teams. Pesce and Montana [11] investigated connectivity-driven communication for multi-agent coordination, while Shetty et al. [12] proposed decentralized connectivity maintenance under motion and sensing uncertainties. However, these methods rarely combine visual connectivity with an explicit and biologically interpretable role-evolution mechanism for heterogeneous encirclement.

Biological collective hunting offers a useful design principle. Wolf-pack and mixed-species hunting studies show that leadership and contribution are not fixed attributes; rather, they emerge from local observation, spatial position, prey response, and the changing contribution of individuals [15]. Goeckner et al. [16] demonstrated that graph-based learning can improve resilient distributed coordination in multi-robot systems. Ning and Xie [13] reviewed adaptive decision-making mechanisms in multi-agent reinforcement learning, while Wang et al. [14] investigated deep-reinforcement-learning-based multi-robot coordination in complex environments. These studies suggest that UAV–USV roles should evolve from accumulated evidence rather than be determined only by instantaneous conditions. In this paper, visual connectivity is therefore treated as a coordination primitive: agents that maintain target-information paths can become relay agents, agents with strong observation and stability can become leaders, and agents with poor information access can search or recover connectivity.

Unlike conventional role-allocation methods based on fixed roles or instantaneous conditions, the proposed mechanism uses accumulated observation, connectivity, stability, and platform information to achieve continuous role evolution. Moreover, visual connectivity is directly incorporated into relay selection and target-information propagation rather than being treated only as a connectivity constraint.

**Figure 1:**
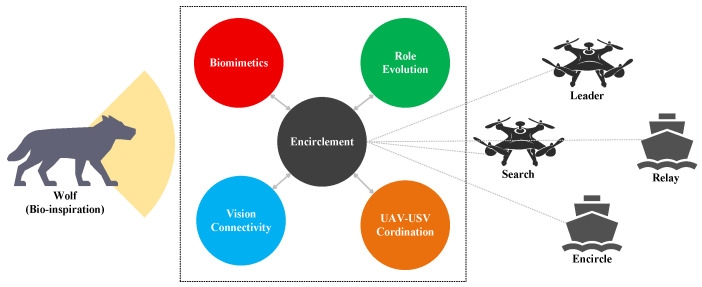
Biologically inspired encirclement mechanism.

Figure 1 summarizes the biological abstraction used in this work. Cai et al. [17] showed that a biologically inspired gene-regulation network can generate emergent UAV-swarm behaviors from local interactions. Shi et al. [18] investigated communication-connectivity restoration in multi-robot systems. Inspired by these studies, the proposed two-layer framework separates role growth and task-state selection from executable motion control, allowing target search, relay maintenance, tracking, and encirclement behaviors to emerge within the same distributed framework.

Compared with representative cooperative encirclement approaches, the principal advantage of GB-VCE lies in extending encirclement from geometric formation regulation to adaptive functional organization. Conventional geometric methods primarily regulate inter-agent distance, bearing, or angular distribution, whereas GB-VCE additionally determines which agents should undertake target guidance, visual relay, search recovery, and geometric enclosure according to their locally evaluated contributions. Unlike fixed-role and static-leader strategies, these functions are not permanently assigned but are reorganized as target observability, visual topology, motion stability, and platform suitability vary. Moreover, the growth-based accumulation of role evidence provides temporal continuity, reducing the sensitivity of instantaneous role-selection schemes to transient sensing and topology fluctuations. In contrast to learning-based coordination, the resulting functional differentiation remains explicit and physically interpretable. Finally, visual connectivity is not treated merely as a feasibility constraint; its contribution is incorporated into role evaluation, so that agents preserving critical target-information paths are actively assigned relay functions. This enables heterogeneous UAVs and USVs to establish complementary roles while maintaining distributed encirclement under constrained communication.

The innovations of this paper are as follows:

1. A wolf-pack-inspired distributed encirclement framework is proposed for heterogeneous UAV–USV teams under communication-constrained visual sensing, enabling cooperative target acquisition, relay maintenance, and encirclement without centralized fusion.
2. A growth-based role-evolution mechanism is developed, in which leader, relay, searcher, and encircler roles emerge from accumulated observation quality, visual connectivity contribution, motion stability, and platform bias.
3. A visual-connectivity-preserving motion-control strategy is designed and validated through multi-seed simulations, showing improved encirclement accuracy, target-information coverage, role stability, and heterogeneous complementarity over baseline methods.

## 2. Related Work

### 2.1. Biomimetic Encirclement and Collective Hunting

Biomimetic coordination seeks transferable principles from biological systems rather than literal imitation. For encirclement, the relevant principles are local perception, progressive spatial closure, role differentiation, and resilient information sharing. Xie et al. [19] reviewed the development and future directions of flocking-control models for multi-agent systems. Yan and Su [20] analyzed the role of alignment interactions in the collective behavior of self-propelled particles. Gable et al. [15] showed that the contribution of individual wolves changes with prey size, prey abundance, and provisioning conditions. Sampaio et al. [21] further demonstrated that leadership and collective success in mixed-species hunting groups depend on social influence and group composition. Oh et al. [22] systematically reviewed multi-agent formation-control methods. Cao et al. [23] summarized the theoretical foundations of distributed multi-agent coordination. Ge et al. [24] reviewed distributed coordination under intermittent sampling and communication. Feng et al. [25] discussed resilient cooperative control and optimization, while Wang et al. [26] investigated resilient consensus control under disturbances and failures. These studies motivate an encirclement mechanism that integrates geometric enclosure, visual connectivity preservation, and temporally continuous role growth. These results motivate an encirclement mechanism that combines geometric enclosure, visual connectivity preservation, and temporally continuous role growth.

### 2.2. Target Enclosing Control and Learning-Based Coordination

Target enclosing has been addressed through bearing control, circular formation control, collision avoidance, and learning-based planning. Chen et al. [1] considered field-of-view constraints and uncertain dynamics in monocular vision-based USV target enclosing. Chen et al. [2] addressed cooperative enclosing using bearing-only measurements under unknown USV dynamics and sideslip. Ma et al. [27] applied deep reinforcement learning to multi-robot target encirclement with collision avoidance. Zhang et al. [28] extended this approach to multi-target encirclement. Lowe et al. [7] developed a multi-agent actor–critic framework for decentralized policy learning. Rashid et al. [8] proposed value-function factorization for cooperative decision making, whereas Foerster et al. [9] introduced counterfactual policy gradients for multi-agent credit assignment. However, the functional division learned by these approaches is generally implicit. Chung et al. [29] reviewed aerial swarm robotics and emphasized the importance of distributed sensing and coordination. Qu et al. [30] applied multi-agent reinforcement learning to cooperative encirclement by autonomous surface vehicles. These studies indicate that sensing and interaction topology are as important as encirclement geometry in air–sea cooperative tasks. The proposed GB-VCE differs by making roles explicit, interpretable, and driven by local visual connectivity evidence.

## 3. Architecture and Mathematical Model

### 3.1. Overall Architecture of the Biological Distributed Encirclement Framework

**Figure 2:**
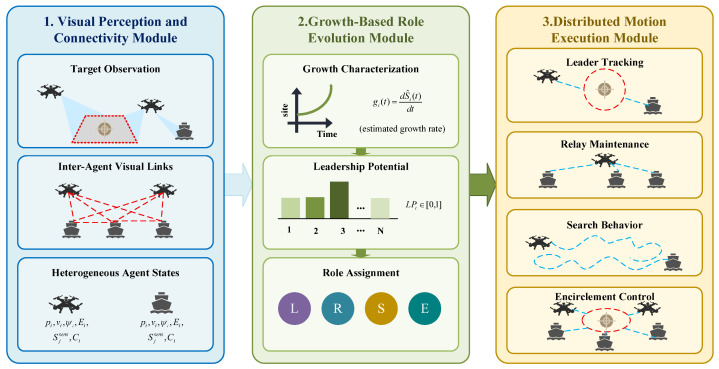
Overall architecture of the proposed UAV–USV biological distributed encirclement framework.

The proposed GB-VCE framework shown in Figure 2 realizes cooperative encirclement of a maneuvering surface target by a heterogeneous UAV–USV team. The architecture contains three coupled modules: visual perception and connectivity evaluation, growth-based role evolution, and role-dependent motion execution. UAVs contribute fast relocation and wide-area visual relay, whereas USVs provide persistent surface-level encirclement. Instead of assigning a permanent leader, the system lets leadership and relay functions emerge from accumulated observation quality, connectivity contribution, and motion stability.

**Figure 3:**
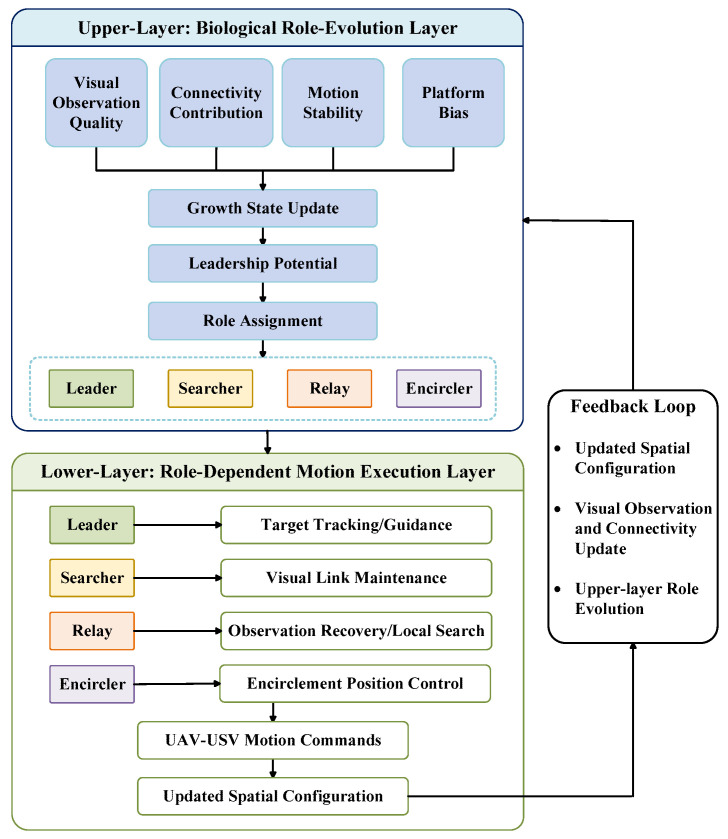
Two-layer distributed decision structure.

The framework follows a two-layer decision structure shown in Figure 3. The upper layer evaluates biological growth state and role potential, while the lower layer transforms the selected role into executable acceleration and heading commands. This separation keeps the method interpretable: the leader guides target-related encirclement, relay agents preserve visual information propagation, searchers recover target or teammate visibility, and encirclers regulate their angular distribution around the target.

At each sampling instant, local measurements update the target observation quality $O_{i}\left(t\right)$, the visual graph $G_{v}\left(t\right)$, and the connectivity contribution $C_{i}\left(t\right)$. These variables drive the growth state $z_{i}\left(t\right)$ and leadership potential $P_{i}\left(t\right)$, after which the role variable is selected as

(1) $$r_{i}\left(t\right)\in \left\{L,R,S,E\right\},$$

where *L*, *R*, *S*, and *E* denote leader, relay, searcher, and encircler. The resulting closed loop is summarized by

(2) $$\left\{O_{i}\left(t\right),G_{v}\left(t\right),C_{i}\left(t\right)\right\}\to \left\{g_{i}\left(t\right),z_{i}\left(t\right),P_{i}\left(t\right)\right\}\to r_{i}\left(t\right)\to \left(a_{i}^{c}\left(t\right),\alpha_{i}^{c}\left(t\right)\right)\to x_{i}\left(t+\Delta t\right).$$

where $g_{i}\left(t\right)$, $z_{i}\left(t\right)$, and $P_{i}\left(t\right)$ denote the growth drive, accumulated growth state, and leadership potential, respectively. This loop links perception, role evolution, and motion execution without centralized state fusion.

### 3.2. Heterogeneous Agent, Target, and Visual Connectivity Model

Let the heterogeneous team be

(3) $$A=A_{\mathrm{UAV}}\cup A_{\mathrm{USV}},\;N=\left|A\right|.$$

For agent *i*, the planar state is denoted by $x_{i}={\left[p_{i}^{⊤},\psi_{i},v_{i},\omega_{i}\right]}^{⊤}$, where $p_{i}\in R^{2}$ is position, $\psi_{i}$ is heading, and $v_{i}$ and $\omega_{i}$ are linear and angular velocities. The target state is $x_{T}={\left[p_{T}^{⊤},v_{T}^{⊤}\right]}^{⊤}$. Heterogeneity is represented by platform-dependent limits on sensing range, field of view, speed, acceleration, and turning rate.

A visual link from agent *i* to object *j* is valid only when range and bearing constraints are both satisfied. With relative vector $d_{ij}=p_{j}-p_{i}$, the distance condition is $∥d_{ij}∥\;\leq R_{i}$, and the field-of-view condition is

(4) $$\left|\mathrm{wrap}\left(\mathrm{atan}2\left(d_{ij,y},d_{ij,x}\right)-\psi_{i}\right)\right|\leq \frac{\Phi_{i}}{2}.$$

Here, wrap(·) maps an angle to the interval (−π,π]. The visual accessibility indicator is therefore

(5) $$\delta_{ij}\left(t\right)=I\left(∥d_{ij}∥\;\leq R_{i},\;\left|\beta_{ij}\left(t\right)\right|\;\leq \Phi_{i}/2\right),$$

and the visual graph is $G_{v}\left(t\right)=\left(A,E_{v}\left(t\right)\right)$, where $\left(i,j\right)\in E_{v}\left(t\right)$ if a bidirectional or task-valid visual relation exists. Target observation quality is normalized as

(6) $$O_{i}\left(t\right)=\delta_{iT}\left(t\right)\exp\left(-\frac{∥p_{i}-p_{T}{∥}^{2}}{\sigma_{R}^{2}}\right)\cos^{+}\;\beta_{iT}\left(t\right),$$

where $\cos^{+}\left(x\right)=\max\left(0,\cos x\right)$. Since all factors in Equation (6) are bounded, the resulting observation-quality index satisfies $0\leq O_{i}\left(t\right)\leq 1$. This normalization provides a dimensionless and comparable measure across heterogeneous UAV and USV sensing conditions, facilitates its integration with the other role-evaluation terms, and supports consistent threshold selection in the subsequent role-evolution process. Connectivity contribution $C_{i}\left(t\right)$ is computed from the visual graph by combining local degree, bridge contribution, and accessibility to target-observing agents. This term rewards agents that preserve the visual information path rather than merely approaching the target.

### 3.3. Growth-Based Role Evolution Model

The biomimetic role mechanism treats leadership as a growth process. The instantaneous growth drive of agent *i* is

(7) $$g_{i}\left(t\right)=w_{O}O_{i}\left(t\right)+w_{C}C_{i}\left(t\right)+w_{S}S_{i}\left(t\right)+w_{H}H_{i},$$

where $O_{i}\left(t\right)$, $C_{i}\left(t\right)$, $S_{i}\left(t\right)$, and $H_{i}$ denote target observation quality, connectivity contribution, motion stability, and heterogeneous platform bias, respectively; $w_{O}$, $w_{C}$, $w_{S}$, and $w_{H}$ are the corresponding nonnegative weights.

(8) $$z_{i}\left(t+\Delta t\right)=\left(1-\lambda_{g}\right)z_{i}\left(t\right)+\Delta t\;g_{i}\left(t\right),$$

where $\lambda_{g}$ is the decay coefficient. This accumulation prevents role changes caused by short visual fluctuations.

Motivated by the observation that leadership and individual contribution in collective hunting vary with local perception, spatial position, and task-dependent contribution [15], and by adaptive role-decision principles in multi-agent systems [13], we define a composite leadership-potential function in this study as

(9) $$P_{i}\left(t\right)=\eta_{z}z_{i}\left(t\right)+\eta_{O}O_{i}\left(t\right)+\eta_{C}C_{i}\left(t\right)-\eta_{D}D_{i}\left(t\right),$$

where $\eta_{z}$, $\eta_{O}$, $\eta_{C}$, and $\eta_{D}$ are weighting coefficients for growth state, observation quality, connectivity contribution, and penalty term, respectively. $D_{i}\left(t\right)$ penalizes excessive distance or unstable motion. The leader is selected by hysteretic maximization

(10) $$L\left(t\right)=\arg\max_{i\in A}P_{i}\left(t\right),\;P_{L(t)}\left(t\right)>P_{L(t^{-})}\left(t\right)+h_{L},$$

where $h_{L}$ is a switching margin. Relay agents are selected from nodes with high $C_{i}\left(t\right)$, searchers are assigned when both observation and connectivity are weak, and the remaining agents are encirclers. This creates explicit and temporally smooth roles, consistent with adaptive biological hunting rather than instantaneous thresholding [13].

### 3.4. Distributed Encirclement Control Model

For an encircler, the desired position is defined as a point on a circle centered at the estimated target position, so that different encirclers occupy different angular slots around the target:

(11) $$p_{i}^{d}\left(t\right)={\hat{p}}_{T}\left(t\right)+R_{E}\left[\begin{array}{c} \cos\theta_{i}^{d}\left(t\right)\\ \sin\theta_{i}^{d}\left(t\right) \end{array}\right],$$

where $R_{E}$ is the desired encirclement radius and $\theta_{i}^{d}$ is generated from the angular distribution of active encirclers. Leader and relay agents use the same target reference but add stronger observation and connectivity terms. Search agents move toward the predicted target region or toward positions that increase visual coverage.

The commanded planar acceleration is written as

(12) $$u_{i}\left(t\right)=u_{i}^{\mathrm{role}}\left(t\right)+u_{i}^{\mathrm{conn}}\left(t\right)+u_{i}^{\mathrm{align}}\left(t\right)+u_{i}^{\mathrm{safe}}\left(t\right),$$

where the four terms correspond to role objective tracking, visual connectivity maintenance, direction alignment, and collision avoidance. The command is then mapped to the heterogeneous execution model subject to platform limits

(13) $$a_{i}^{c}={\mathrm{sat}}_{a_{i}}\left(u_{i}^{⊤}e_{\psi_{i}}\right),\;\alpha_{i}^{c}={\mathrm{sat}}_{\alpha_{i}}\left(k_{\psi }\;\mathrm{wrap}\left(\psi_{i}^{d}-\psi_{i}\right)-k_{\omega }\omega_{i}\right).$$

The feasibility of encirclement is evaluated by success rate, radius error, stable encirclement time, target-information coverage, role switching frequency, and control smoothness. These metrics jointly assess spatial enclosure, visual information preservation, and biological role stability.

## 4. Methods

GB-VCE comprises three complementary modules for distributed heterogeneous encirclement. The connectivity-aware task-state switching module regulates the transition among target acquisition, connectivity recovery, and cooperative encirclement according to target-information availability, thereby avoiding premature coordination based on incomplete observations. The growth-based role-evolution module adaptively organizes agents into leader, relay, searcher, and encircler roles using accumulated local evidence, providing greater adaptability than fixed role assignment and greater temporal stability than instantaneous selection. The role-dependent control module maps these functional roles to platform-specific motion objectives, enabling UAVs and USVs to exploit their complementary sensing and mobility capabilities more effectively than a unified control strategy. Together, these modules improve information consistency, role reconfiguration, and heterogeneous coordination compared with simpler methods that rely on direct encirclement, static roles, or identical agent behaviors.

### 4.1. Target Acquisition and Connectivity-Aware Task-State Switching

In heterogeneous pursuit–encirclement missions, target visibility and information accessibility may be intermittent. Therefore, GB-VCE first uses a connectivity-aware task-state switch to distinguish target acquisition, connectivity recovery, and cooperative encirclement. Full encirclement is activated only when at least one valid target observation exists and target-related information is accessible to a sufficient portion of the swarm.

The three task states are defined as follows: target acquisition when no agent observes the target, connectivity recovery when the target is observed but visual information has not propagated sufficiently, and cooperative encirclement when observation and accessibility conditions are both satisfied.

Let A={1,2,…,N} denote the heterogeneous team. An observation by agent *i* is effective when $O_{i}\left(t\right)>\epsilon_{O}$, yielding

(14) $$S_{T}\left(t\right)=\left\{i\in A|O_{i}\left(t\right)>\epsilon_{O}\right\}$$

A binary target-availability variable is then introduced as

(15) $$\zeta_{T}\left(t\right)=\left\{\begin{array}{cc} 1, & S_{T}\left(t\right)\neq ⌀,\\ 0, & S_{T}\left(t\right)=⌀. \end{array}\right.$$

Here, $\zeta_{T}\left(t\right)$ indicates whether valid target information is currently available.

A single isolated observation is insufficient for collective encirclement; therefore, target-information accessibility is evaluated on the visual graph. Let $A_{\mathrm{vis}}\left(t\right)$ denote the visual connectivity adjacency matrix. For agent *i*, the accessibility indicator $\xi_{i}\left(t\right)$ is defined as

(16) $$\xi_{i}\left(t\right)=\left\{\begin{array}{cc} 1, & \mathrm{agent}\;i\;\mathrm{is}\;\mathrm{connected}\;\mathrm{to}\;\mathrm{at}\;\mathrm{least}\;\mathrm{one}\;\mathrm{agent}\;\mathrm{in}\;S_{T}\left(t\right)\;\mathrm{through}\;A_{\mathrm{vis}}\left(t\right),\\ 0, & \mathrm{otherwise}. \end{array}\right.$$

Based on $\xi_{i}\left(t\right)$, the target-information coverage ratio of the swarm is defined as

(17) $$\Gamma_{T}\left(t\right)=\frac{1}{N}\sum_{i=1}^{N}\xi_{i}\left(t\right)$$

$\Gamma_{T}\left(t\right)$ measures the fraction of agents with direct or relayed access to target information.

The control layer uses an estimated target reference. When the target is visible, the estimate is updated as

(18) $${\hat{p}}_{T}\left(t\right)=p_{T}\left(t\right),\;{\hat{v}}_{T}\left(t\right)=v_{T}\left(t\right),\;\mathrm{if}\;\zeta_{T}\left(t\right)=1$$

and the latest observation time is recorded as $t_{\mathrm{last}}=t$. When the target is temporarily lost but has been observed previously, a constant-velocity prediction model is used

(19) $${\hat{p}}_{T}\left(t\right)={\hat{p}}_{T}\left(t-\Delta t\right)+\Delta t\;{\hat{v}}_{T}\left(t-\Delta t\right),\;\mathrm{if}\;\zeta_{T}\left(t\right)=0,\;t-t_{\mathrm{last}}\leq T_{\mathrm{lost}}$$

where Δt is the sampling interval and $T_{\mathrm{lost}}$ is the maximum allowable duration for short-term target-state prediction. This treatment allows the agents to continue searching around the most probable target region after a temporary loss of visual contact.

If the target has never been observed, or if the target has been lost for longer than $T_{\mathrm{lost}}$, the extrapolated target estimate is no longer regarded as reliable. In this case, the target reference is reset to the global search center $p_{c}$, and the estimated velocity is set to zero

(20) $${\hat{p}}_{T}\left(t\right)=p_{c},\;{\hat{v}}_{T}\left(t\right)=0,\;\mathrm{if}\;\zeta_{T}\left(t\right)=0,\;t-t_{\mathrm{last}}>T_{\mathrm{lost}}$$

This reset prevents pursuit of unreliable extrapolations and reorganizes the team around a distributed search pattern.

Each agent uses an information-consistent target reference:

(21) $$p_{T,i}^{c}\left(t\right)=\left\{\begin{array}{cc} p_{T}\left(t\right), & \xi_{i}\left(t\right)=1,\\ {\hat{p}}_{T}\left(t\right), & \xi_{i}\left(t\right)=0, \end{array}\right.$$

and

(22) $$v_{T,i}^{c}\left(t\right)=\left\{\begin{array}{cc} v_{T}\left(t\right), & \xi_{i}\left(t\right)=1,\\ {\hat{v}}_{T}\left(t\right), & \xi_{i}\left(t\right)=0. \end{array}\right.$$

Thus, disconnected agents use predicted references instead of unavailable true target states. The task state is jointly determined by $\zeta_{T}\left(t\right)$ and $\Gamma_{T}\left(t\right)$. If $\zeta_{T}\left(t\right)=0$, leader election is suspended and all agents search.

The threshold $\Gamma_{\mathrm{th}}$ determines whether dissemination is sufficient. If $\zeta_{T}\left(t\right)=1$ but $\Gamma_{T}\left(t\right)<\Gamma_{\mathrm{th}}$, the team enters connectivity recovery and selects the strongest observer

(23) $$i_{L}\left(t\right)=\arg\max_{i\in S_{T}\left(t\right)}O_{i}\left(t\right)$$

The observing leader maintains target contact, while other agents execute relay or search motions until visual information becomes sufficiently accessible.

If $\zeta_{T}\left(t\right)=1$ and $\Gamma_{T}\left(t\right)\geq \Gamma_{\mathrm{th}}$, the cooperative-encirclement state activates the growth-based role assignment.

The complete target acquisition and connectivity-aware task-state switching procedure is summarized in Algorithm 1.


**Algorithm 1** Connectivity-aware target acquisition and task-state switching.
**Require:** 
A, ${\left\{O_{i}\left(t\right)\right\}}_{i=1}^{N}$, $\epsilon_{O}$, $A_{\mathrm{vis}}\left(t\right)$, $\Gamma_{\mathrm{th}}$, $\left({\hat{p}}_{T},{\hat{v}}_{T}\right)$, $t_{\mathrm{last}}$, $T_{\mathrm{lost}}$, $p_{c}$**Ensure:** 
M(t), ${\left\{\left(p_{T,i}^{c},v_{T,i}^{c}\right)\right\}}_{i=1}^{N}$, ${\left\{r_{i}\left(t\right)\right\}}_{i=1}^{N}$, leader candidate $i_{L}\left(t\right)$  1:

$S_{T}\left(t\right)\leftarrow \left\{i\in A|O_{i}\left(t\right)>\epsilon_{O}\right\}$

  2:Compute $\xi_{i}\left(t\right)$ from $A_{\mathrm{vis}}\left(t\right)$ and $S_{T}\left(t\right)$; set $\Gamma_{T}\left(t\right)\leftarrow N^{-1}\sum_{i=1}^{N}\xi_{i}\left(t\right)$  3:**if** $S_{T}\left(t\right)=⌀$ **then**  4:    $\zeta_{T}\left(t\right)\leftarrow 0$, M(t)← target acquisition state  5:    Suspend leader election; assign $r_{i}\left(t\right)\leftarrow R_{S}$, ∀i∈A  6:    **if** target has been observed before and $t-t_{\mathrm{last}}\leq T_{\mathrm{lost}}$ **then**  7:        ${\hat{p}}_{T}\left(t\right)\leftarrow {\hat{p}}_{T}\left(t-\Delta t\right)+\Delta t\;{\hat{v}}_{T}\left(t-\Delta t\right)$  8:    **else**  9:        ${\hat{p}}_{T}\left(t\right)\leftarrow p_{c}$, ${\hat{v}}_{T}\left(t\right)\leftarrow 0$10:    **end if**11:    Set $\left(p_{T,i}^{c}\left(t\right),v_{T,i}^{c}\left(t\right)\right)\leftarrow \left({\hat{p}}_{T}\left(t\right),{\hat{v}}_{T}\left(t\right)\right)$, ∀i∈A12:
**else**
13:    $\zeta_{T}\left(t\right)\leftarrow 1$14:    ${\hat{p}}_{T}\left(t\right)\leftarrow p_{T}\left(t\right)$, ${\hat{v}}_{T}\left(t\right)\leftarrow v_{T}\left(t\right)$, $t_{\mathrm{last}}\leftarrow t$15:    Set $\left(p_{T,i}^{c}\left(t\right),v_{T,i}^{c}\left(t\right)\right)\leftarrow \left(p_{T}\left(t\right),v_{T}\left(t\right)\right)$ for agents with $\xi_{i}\left(t\right)=1$, and $\left({\hat{p}}_{T}\left(t\right),{\hat{v}}_{T}\left(t\right)\right)$ for agents with $\xi_{i}\left(t\right)=0$16:    $i_{L}\left(t\right)\leftarrow \arg\max_{i\in S_{T}\left(t\right)}O_{i}\left(t\right)$17:    **if** $\Gamma_{T}\left(t\right)<\Gamma_{\mathrm{th}}$ **then**18:        M(t)← connectivity-recovery state19:        Assign $r_{i_{L}}\left(t\right)\leftarrow R_{L}$; assign non-leader agents to relay-oriented or search-oriented recovery20:    **else**21:        M(t)← cooperative-encirclement state22:        Activate growth-based adaptive role assignment23:    **end if**24:
**end if**
25:**return** M(t), ${\left\{\left(p_{T,i}^{c}\left(t\right),v_{T,i}^{c}\left(t\right)\right)\right\}}_{i=1}^{N}$, ${\left\{r_{i}\left(t\right)\right\}}_{i=1}^{N}$, $i_{L}\left(t\right)$



### 4.2. Growth-Based Adaptive Role Assignment

Once cooperative encirclement is active, agents are assigned to functional roles according to accumulated growth evidence rather than fixed labels.

The growth state integrates observation quality, connectivity contribution, stability, and platform bias, thereby filtering transient sensing errors and topology fluctuations.

Leader selection uses accumulated leadership potential with switching margin and holding-time constraints. Relay agents preserve visual information paths, searchers recover weak observation/connectivity, and the remaining agents form the encirclement ring.

In Algorithm 2, the auxiliary variable ${\tilde{g}}_{i}\left(t\right)$ denotes the instantaneous growth drive before temporal smoothing. The relay score ΨiRe(t) evaluates whether agent *i* is suitable for maintaining visual information paths. Here, $d_{iL}\left(t\right)=∥p_{i}\left(t\right)-p_{i_{L}}\left(t\right)∥$ and $d_{iT}\left(t\right)=∥p_{i}\left(t\right)-p_{T,i}^{c}\left(t\right)∥$ denote the distances from agent *i* to the current leader and to its target reference, respectively. NRe is the prescribed number of relay agents selected from the remaining agents according to the relay score. The variables $\tau_{i}^{S}\left(t\right)$ and $\sigma_{i}^{S}\left(t\right)$ denote the duration of poor observation/connectivity and the binary searcher-state flag, respectively. The thresholds $O_{\mathrm{low}}$, $O_{\mathrm{rec}}$, $C_{\mathrm{low}}^{T}$, and $C_{\mathrm{rec}}^{T}$ define the entry and recovery conditions for the searcher role, where $C_{i}^{T}\left(t\right)$ measures the local accessibility of target-related information. The sets $A_{\mathrm{rem}}$, ARe, $A_{\mathrm{res}}$, $A_{S}$, and $A_{E}$ denote the remaining agents after leader selection, selected relay agents, residual agents after relay selection, searchers, and encirclers, respectively.
**Algorithm 2** Growth-based adaptive role assignment.**Require:** 
M(t), A, $i_{L}\left(t-\Delta t\right)$, ${\left\{r_{i}\left(t-\Delta t\right)\right\}}_{i=1}^{N}$, ${\left\{g_{i}\left(t-\Delta t\right)\right\}}_{i=1}^{N}$, ${\left\{P_{i}\left(t-\Delta t\right)\right\}}_{i=1}^{N}$**Ensure:** 
${\left\{r_{i}\left(t\right)\right\}}_{i=1}^{N}$, $i_{L}\left(t\right)$  1:**if** $M\left(t\right)\neq M_{\mathrm{CE}}$ **then**  2:    ${\left\{r_{i}\left(t\right)\right\}}_{i=1}^{N}\leftarrow {\left\{r_{i}\left(t-\Delta t\right)\right\}}_{i=1}^{N}$,    $i_{L}\left(t\right)\leftarrow i_{L}\left(t-\Delta t\right)$  3:    **return** ${\left\{r_{i}\left(t\right)\right\}}_{i=1}^{N},i_{L}\left(t\right)$  4:**end if**  5:**for** i=1 to *N* **do**  6:    ${\tilde{g}}_{i}\left(t\right)\leftarrow f_{g}\left(O_{i}\left(t\right),C_{i}\left(t\right),S_{i}\left(t\right),H_{i}\right)$  7:    $g_{i}\left(t\right)\leftarrow \lambda_{g}g_{i}\left(t-\Delta t\right)+\left(1-\lambda_{g}\right){\tilde{g}}_{i}\left(t\right)$  8:    $P_{i}\left(t\right)\leftarrow f_{L}\left(g_{i}\left(t\right),O_{i}\left(t\right),C_{i}\left(t\right),S_{i}\left(t\right),H_{i}\right)$  9:**end for**10:$i_{L}^{\mathrm{cand}}\leftarrow \arg\max_{i\in A}P_{i}\left(t\right)$,    $\Delta P_{L}\leftarrow P_{i_{L}^{\mathrm{cand}}}\left(t\right)-P_{i_{L}\left(t-\Delta t\right)}\left(t\right)$11:$i_{L}\left(t\right)\leftarrow \left\{\begin{array}{cc} i_{L}^{\mathrm{cand}}, & \Delta P_{L}\geq \Delta P_{\mathrm{th}}\;\mathrm{and}\;T_{L}\left(t\right)\geq T_{L}^{\min},\\ i_{L}\left(t-\Delta t\right), & \mathrm{otherwise}. \end{array}\right.$12:$r_{i_{L}\left(t\right)}\left(t\right)\leftarrow R_{L}$,    $A_{\mathrm{rem}}\leftarrow A\setminus \left\{i_{L}\left(t\right)\right\}$13:**for** $i\in A_{\mathrm{rem}}$ **do**14:   ΨiRe(t)←fReCi(t),diL(t),diT(t),Oi(t)15:**end for**16:ARe←TopKi∈AremΨiRe(t),NRe17:Ares←Arem∖ARe18:**for** $i\in A_{\mathrm{res}}$ **do**19:    $\tau_{i}^{S}\left(t\right)\leftarrow \left\{\begin{array}{cc} \tau_{i}^{S}\left(t-\Delta t\right)+\Delta t, & O_{i}\left(t\right)<O_{\mathrm{low}}\;\mathrm{and}\;C_{i}^{T}\left(t\right)<C_{\mathrm{low}}^{T},\\ 0, & \mathrm{otherwise}. \end{array}\right.$20:    $\sigma_{i}^{S}\left(t\right)\leftarrow \left\{\begin{array}{cc} 1, & \tau_{i}^{S}\left(t\right)\geq T_{S}^{\mathrm{in}},\\ 0, & O_{i}\left(t\right)\geq O_{\mathrm{rec}}\;\mathrm{or}\;C_{i}^{T}\left(t\right)\geq C_{\mathrm{rec}}^{T},\\ \sigma_{i}^{S}\left(t-\Delta t\right), & \mathrm{otherwise}. \end{array}\right.$21:**end for**22:$A_{S}\leftarrow \left\{i\in A_{\mathrm{res}}|\sigma_{i}^{S}\left(t\right)=1\right\}$,    $A_{E}\leftarrow A_{\mathrm{res}}\setminus A_{S}$23:ri(t)←RRe,∀i∈ARe;    $r_{i}\left(t\right)\leftarrow R_{S},\;\forall i\in A_{S}$;    $r_{i}\left(t\right)\leftarrow R_{E},\;\forall i\in A_{E}$24:**return** ${\left\{r_{i}\left(t\right)\right\}}_{i=1}^{N},i_{L}\left(t\right)$

The output of this module is not a direct motion command, but a role label for each agent. These role labels are passed to the role-dependent motion-control module in Section 4.3, where they are converted into executable motion objectives. The complete procedure is summarized in Algorithm 2.

### 4.3. Role-Dependent Motion Control

The role-dependent motion-control module converts the task state and adaptive role assignment into physically feasible motion commands for the heterogeneous UAV–USV swarm. In contrast to a fixed formation controller, the proposed controller does not impose an identical motion objective on all agents. Instead, each agent generates its motion command according to its current biological role, locally accessible target reference, visual-neighbor topology, and platform-dependent dynamic constraints. This design allows target guidance, information relay, search recovery, and encirclement formation to be executed within a unified distributed control process.

At each control step, the current role selects a role-specific virtual velocity: target guidance for the leader, information-path maintenance for relays, exploration for searchers, and ring tracking for encirclers. Agent-wise target references ensure distributed information consistency.

For agent *i*, the heading-alignment term is obtained by averaging the unit heading vectors of its currently visible neighbors:

(24) $${\bar{v}}_{i}\left(t\right)=\frac{1}{|N_{i}^{v}\left(t\right)|}\sum_{j\in N_{i}^{v}\left(t\right)}\left[\begin{array}{c} \cos\psi_{j}\left(t\right)\\ \sin\psi_{j}\left(t\right) \end{array}\right],$$

where $N_{i}^{v}\left(t\right)$ denotes the visual-neighbor set of agent *i*. If no visual neighbor is available, ${\bar{v}}_{i}\left(t\right)$ is set to zero. The term $k_{\mathrm{align}}{\bar{v}}_{i}\left(t\right)$ is added to the role-dependent desired velocity to improve local directional consistency without overriding the primary role objective.

Connectivity-maintenance and heading-alignment corrections are then added before bounded acceleration commands are generated under UAV/USV-specific limits.

Algorithm 3 summarizes the role-dependent controller.
**Algorithm 3** Role-dependent motion control.**Require:** 
Agent states ${\left\{p_{i},\psi_{i},v_{i},\omega_{i}\right\}}_{i=1}^{N}$, roles ${\left\{r_{i}\right\}}_{i=1}^{N}$, target references ${\left\{\left(p_{T,i}^{c},v_{T,i}^{c}\right)\right\}}_{i=1}^{N}$, visual graph $A_{\mathrm{vis}}$, leader $i_{L}$, platform limits**Ensure:** 
Control inputs ${\left\{a_{i}^{c},\alpha_{i}^{c}\right\}}_{i=1}^{N}$ and updated states  1:**for** i=1 to *N* **do**  2:    Compute $r_{iT}\leftarrow p_{i}-p_{T,i}^{c}$, $d_{iT}\leftarrow ∥r_{iT}∥$, $e_{r,i}\leftarrow r_{iT}/\left(d_{iT}+\varepsilon \right)$, $e_{t,i}\leftarrow {\left[-e_{r,i}^{y},e_{r,i}^{x}\right]}^{⊤}$  3:    **if** $r_{i}=R_{L}$ **then**  4:        $p_{L}^{d}\leftarrow p_{T,i}^{c}+\gamma_{L}R_{E}{\left[-v_{T,i}^{c,y},v_{T,i}^{c,x}\right]}^{⊤}/(∥v_{T,i}^{c}∥+\varepsilon )$  5:        $v_{i,\mathrm{role}}^{\mathrm{ref}}\leftarrow k_{L}\left(p_{L}^{d}-p_{i}\right)+k_{LT}v_{T,i}^{c}+k_{Lo}e_{t,i}$  6:    **else if** ri=RRe **then**  7:        $p_{\mathrm{mid}}\leftarrow \lambda_{L}p_{i_{L}}+\left(1-\lambda_{L}\right)p_{T,i}^{c}$  8:        $v_{i,\mathrm{role}}^{\mathrm{ref}}\leftarrow k_{Re}\left(p_{\mathrm{mid}}-p_{i}\right)+k_{\mathrm{conn}}v_{i}^{\mathrm{conn}}+k_{RT}v_{T,i}^{c}$  9:    **else if** $r_{i}=R_{S}$ **then**10:        $\theta_{i}^{S}\leftarrow \omega_{S}t+\phi_{i}$, $p_{i}^{S}\leftarrow p_{T,i}^{c}+r_{S}{\left[\cos\theta_{i}^{S},\sin\theta_{i}^{S}\right]}^{⊤}$11:        $v_{i,\mathrm{role}}^{\mathrm{ref}}\leftarrow k_{S1}\left(p_{i}^{S}-p_{i}\right)+k_{S2}\left(p_{T,i}^{c}-p_{i}\right)$12:    **else**13:        $\varphi_{i}^{d}\leftarrow 2\pi k_{i}/N_{E}$, $p_{i}^{E}\leftarrow p_{T,i}^{c}+R_{E}{\left[\cos\varphi_{i}^{d},\sin\varphi_{i}^{d}\right]}^{⊤}$14:        $v_{i,\mathrm{role}}^{\mathrm{ref}}\leftarrow k_{E}\left(p_{i}^{E}-p_{i}\right)+k_{o}e_{t,i}+k_{ET}v_{T,i}^{c}$15:    **end if**16:    $N_{i}^{v}\leftarrow \left\{j\in A\setminus \left\{i\right\}|A_{\mathrm{vis},ij}=1\right\}$17:    Compute $v_{i}^{\mathrm{cm}}$ from pairwise visual-neighbor distance regulation, and compute ${\bar{v}}_{i}$ from average neighbor heading alignment18:    $v_{i}^{\mathrm{des}}\leftarrow v_{i,\mathrm{role}}^{\mathrm{ref}}+k_{\mathrm{cm}}v_{i}^{\mathrm{cm}}+k_{\mathrm{align}}{\bar{v}}_{i}$19:    $s_{i}\leftarrow ∥v_{i}^{\mathrm{des}}∥$, $\psi_{i}^{\mathrm{ref}}\leftarrow \mathrm{atan}2\left(v_{i,y}^{\mathrm{des}},v_{i,x}^{\mathrm{des}}\right)$, $e_{\psi ,i}\leftarrow \mathrm{wrap}\left(\psi_{i}^{\mathrm{ref}}-\psi_{i}\right)$20:    $v_{i}^{\mathrm{ref}}\leftarrow \min\left(v_{\chi_{i}}^{\max},s_{i}\right)\max\left(\eta_{\min},\cos e_{\psi ,i}\right)$21:    $\omega_{i}^{\mathrm{ref}}\leftarrow \mathrm{sat}\left(k_{\psi }^{\mathrm{ref}}e_{\psi ,i},-\omega_{\chi_{i}}^{\max},\omega_{\chi_{i}}^{\max}\right)$22:    $a_{i}^{c}\leftarrow \mathrm{sat}\left(k_{v}\left(v_{i}^{\mathrm{ref}}-v_{i}\right),a_{\chi_{i}}^{\min},a_{\chi_{i}}^{\max}\right)$23:    $\alpha_{i}^{c}\leftarrow \mathrm{sat}\left(k_{\omega }\left(\omega_{i}^{\mathrm{ref}}-\omega_{i}\right),-\alpha_{\chi_{i}}^{\max},\alpha_{\chi_{i}}^{\max}\right)$24:    Apply velocity-boundary protection to $a_{i}^{c}$ and $\alpha_{i}^{c}$25:    Update $x_{i}$, $y_{i}$, $\psi_{i}$, $v_{i}$, and $\omega_{i}$ using the discrete-time second-order motion model26:**end for**27:**return** ${\left\{a_{i}^{c},\alpha_{i}^{c}\right\}}_{i=1}^{N}$ and updated states

## 5. Experimental Setup

### 5.1. Simulation Environment

All simulations were implemented in MATLAB 2023a on a local workstation equipped with an Intel R12 12700H CPU, 16 GB of RAM, and an NVIDIA RTX 3070 GPU. Table 2 lists the environment and platform parameters.

### 5.2. Algorithm Parameters

The key motion-control and role-evolution parameters are listed in Table 3 and Table 4. To improve reproducibility, the evaluated ranges and selection rationale are provided for the parameters that most directly affect convergence, visual connectivity preservation, and role stability.

The experimental tuning was performed in a staged manner. The motion-control gains were tuned while keeping the role-evolution parameters fixed. The objective of this stage was to ensure that agents could follow their role-dependent reference motions without obvious oscillation or excessive control effort. The role-evolution parameters were tuned while using the selected motion-control gains. This stage focused on reducing transient role switching and maintaining sufficient target-information coverage. The combined parameter set was checked through preliminary paired-seed simulations under the same randomized initial perturbations used in the main experiments.

The final parameters were selected according to four criteria: stable encirclement success, radius error, target-information coverage, and role switching stability. Parameter settings that improved one metric but caused severe degradation in another metric were not used. For example, excessively large tracking gains accelerated convergence but increased oscillatory motion, while overly small leader-switching margins improved responsiveness but caused frequent leader changes.

In general, larger tracking gains accelerate convergence but may increase control effort and oscillatory motion. Smaller tracking gains improve smoothness but delay stable encirclement. A larger growth decay coefficient, switching margin, or holding time reduces frequent role changes, but may slow adaptation to visual-topology changes. The observation and connectivity weights determine the balance between target tracking and information preservation: excessive observation weighting tends to favor target-facing agents, whereas excessive connectivity weighting may delay spatial encirclement by overemphasizing relay behavior. Therefore, the selected values represent a trade-off among convergence speed, radius accuracy, visual connectivity preservation, and role stability.

### 5.3. Evaluation Metrics

We evaluate the performance of each method using the following metrics:

- **Final success metric**: The composite encirclement score at the final simulation step.
- **Mean success score in the last 20 steps**: The average composite score during the final stage of each run.
- **Post-stabilization mean score**: The average composite score after stable encirclement is first reached.
- **Radius error**: The deviation between the achieved encirclement radius and the desired radius.
- **Stable encirclement time**: The time required for the team to first reach stable encirclement.
- **Stable reached rate**: The proportion of runs in which stable encirclement is successfully achieved.
- **Coverage ratio**: The fraction of agents with direct or relayed access to target information.
- **Visual connectivity**: The average strength of valid visual links in the team.
- **Geometric score**: A normalized score measuring radius accuracy and angular distribution.
- **Information score**: A normalized score measuring target-information availability within the team.
- **Role switches**: The total number of role changes during one run.
- **Leader switches**: The number of leader changes during one run.
- **Control effort**: The accumulated motion-control cost during the simulation.
- **Composite value index**: An overall normalized index combining convergence, radius accuracy, information availability, visual connectivity, and control effort.
- **Mean rank and best count**: Ranking-based indicators used to compare UAV-only, USV-only, and UAV–USV configurations across multiple metrics.

### 5.4. Statistical Significance Analysis

To verify whether the observed performance gains were statistically reliable, a paired Wilcoxon signed-rank test was conducted. Since all compared methods were evaluated under identical random seeds and perturbed initial conditions, the results form paired samples. The significance level was set to p<0.05.

The test focuses on the comparison between GB-VCE and the static-leader baseline, because static-leader is the stronger baseline and reaches stable encirclement in most runs, whereas fixed-role fails to reach stable encirclement in all 100 runs. The tested metrics include final success metric, stable encirclement time, final radius error, post-stabilization target-information coverage, and mean information score.

## 6. Results and Performance Analysis

### 6.1. Computational-Time Analysis

The computational cost of the proposed GB-VCE framework was evaluated in terms of wall-clock simulation time. All simulations were executed on the workstation described above, and the reported values were obtained from 100 independent runs under identical experimental settings. In the strategy-comparison study, the average computational times of the fixed-role, static-leader, and GB-VCE models were 1.1371±0.1591 s, 2.0811±0.3374 s, and 1.5198±0.2040 s per 200 s simulated mission, respectively. The corresponding average computation time of GB-VCE was approximately 1.518 ms per control step. In the heterogeneous-value study, the UAV-only, USV-only, and heterogeneous UAV–USV configurations required 1.9405±0.3426 s, 1.4383±0.2609 s, and 1.3390±0.1754 s per run, respectively. In all evaluated cases, the average computation time per control step remained substantially below the adopted sampling interval of 0.2 s. These results indicate that the computational burden of GB-VCE is compatible with online execution under the tested simulation settings.

Table 5 summarizes the computational time of the evaluated simulation studies over 100 independent runs.

### 6.2. Encirclement Performance Comparison

The proposed GB-VCE method was evaluated against fixed-role and static-leader baselines using 100 independent paired-seed runs. The use of 100 runs reduces the influence of random initial positions and target-motion perturbations and provides a sufficiently large sample for reliable statistical comparison. For each seed, all compared methods were evaluated under the same perturbed initial conditions, so the observed differences mainly reflect the coordination strategy rather than random variation.

Table 6 shows that GB-VCE achieves the strongest spatial encirclement performance. It obtains the highest final success metric (0.644±0.060), the highest post-stabilization score (0.640±0.009), and the lowest final radius error (1.040±1.034 m). Compared with the static-leader baseline, GB-VCE reduces stable encirclement time from 68.29±41.15 s to 40.65±11.25 s and reaches stability in all 100 runs, indicating faster and more consistent convergence.

Table 7 further explains the source of this improvement. GB-VCE achieves the highest final coverage ratio (0.726±0.066) and post-stabilization coverage (0.692±0.018), and substantially improves the mean information score in the last 20 steps from 0.490±0.044 for static-leader to 0.716±0.016. Although its role switches are higher than in fixed-role, fixed-role never reaches stable encirclement; compared with static-leader, GB-VCE reduces role switches from 273.96±54.53 to 130.27±27.71.

Table 8 reports the paired Wilcoxon signed-rank test results between GB-VCE and the static-leader baseline. The results show that GB-VCE achieves statistically significant improvements in the main performance dimensions. Specifically, GB-VCE improves the final success metric from 0.5978 to 0.6435 ($p=6.81\times 10^{-3}$), reduces the stable encirclement time from 68.29 s to 40.85 s ($p=1.22\times 10^{-13}$), and decreases the final radius error from 2.8505 m to 1.0397 m ($p=1.45\times 10^{-12}$). In addition, GB-VCE significantly improves post-stabilization target-information coverage and mean information score. These results confirm that the observed performance gains are statistically supported rather than caused by random variations.

#### Representative Dynamics and Statistical Dispersion

**Figure 4:**
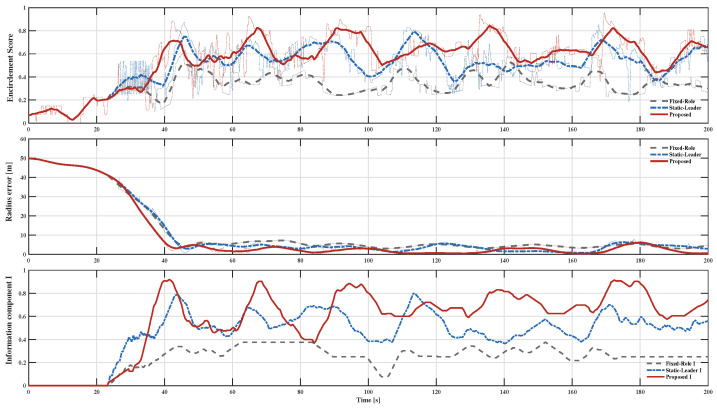
Temporal evolution of representative encirclement-performance metrics for the fixed-role, static-leader, and proposed GB-VCE methods over paired-seed simulations.

**Figure 5:**
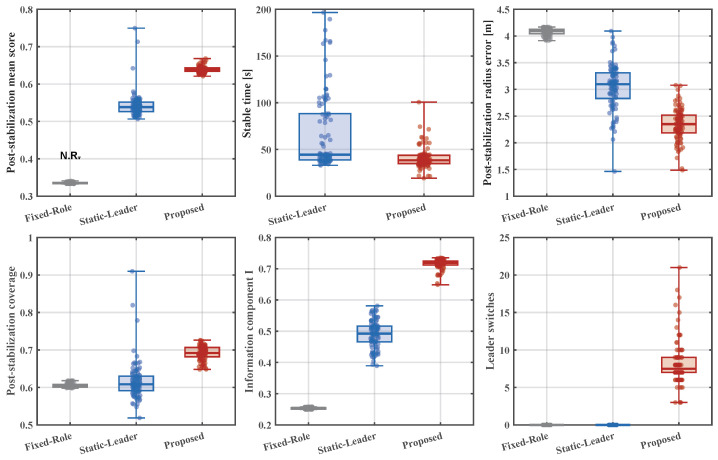
Box-scatter distributions of final success score, stable encirclement time, radius error, information coverage, visual connectivity, and control-related metrics for the compared methods.

**Figure 6:**
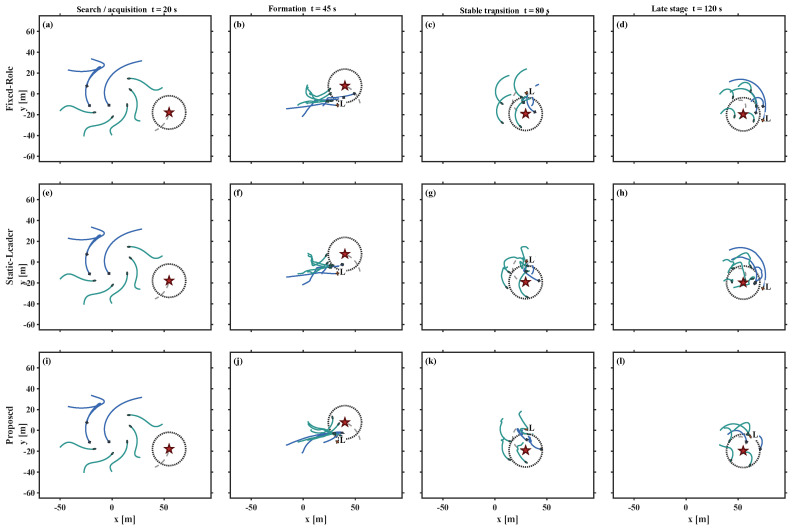
Representative encirclement snapshots of fixed-role (**a**–**d**), static-leader (**e**–**h**), and GB-VCE (**i**–**l**) methods at different mission stages.

**Figure 7:**
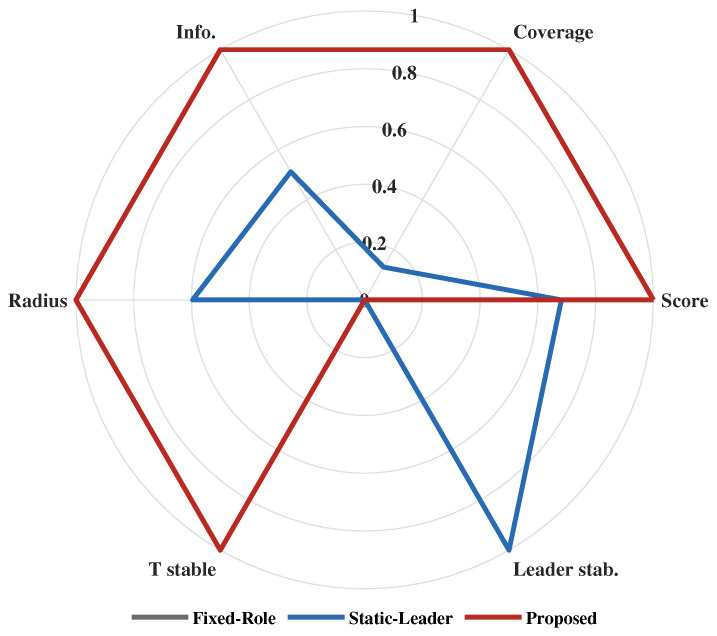
Normalized radar comparison of the fixed-role, static-leader, and proposed GB-VCE methods across success score, radius accuracy, information coverage, role stability, visual connectivity, and control smoothness.

**Figure 8:**
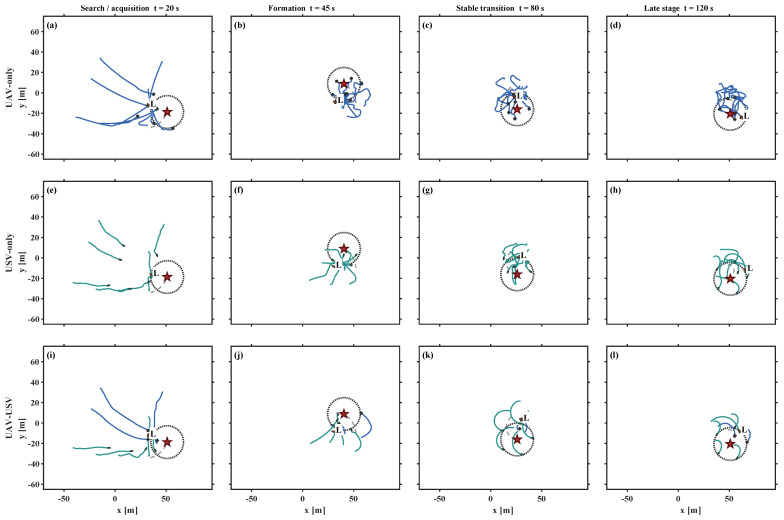
Recovery-to-encirclement snapshots showing visual relay formation and target-information propagation. UAV-only (**a**–**d**), USV-only (**e**–**h**) and UAV-USV (**i**–**l**).

Figure 4, Figure 5, Figure 6, Figure 7 and Figure 8 visualize the same trend from time-series, distributional, and snapshot perspectives.

The performance curves in Figure 4 show that GB-VCE rises faster and stabilizes at a higher score than the baselines. The fixed-role method remains limited by its inability to redistribute sensing and relay functions, whereas the static-leader method improves convergence but is more sensitive to initial visibility and leader placement.

The box-scatter results in Figure 5 indicate that GB-VCE has both higher median performance and smaller dispersion, especially for final success and radius-error metrics. This supports the conclusion that growth-based role evolution improves not only average performance but also run-to-run reliability.

Figure 6 qualitatively shows the formation process, while the quantitative results in Table 6 and Table 7 confirm the smaller final radius error, higher geometric score, and better information coverage of GB-VCE. GB-VCE forms a tighter and more symmetric encirclement ring around the target than the fixed-role and static-leader baselines.

The normalized radar comparison in Figure 7 illustrates the trade-off among success score, radius accuracy, information coverage, role stability, and control smoothness. GB-VCE provides the most balanced profile, which is important for heterogeneous UAV–USV deployment where a single metric cannot capture mission quality.

### 6.3. Heterogeneous Value Verification

To isolate the value of platform heterogeneity, the proposed role-evolution mechanism was also tested with UAV-only, USV-only, and UAV–USV teams under the same number of agents and paired seeds.

Figure 8 compares the recovery-to-encirclement process for UAV-only, USV-only, and heterogeneous UAV–USV teams. UAV-only teams are fast and agile, bridging information gaps quickly but with less stable coverage. USV-only teams maintain stable encirclement but converge slowly. The heterogeneous UAV–USV team combines both strengths: UAVs act as fast information relays, while USVs provide a stable encirclement backbone, achieving both rapid convergence and robust, complete coverage.

**Figure 9:**
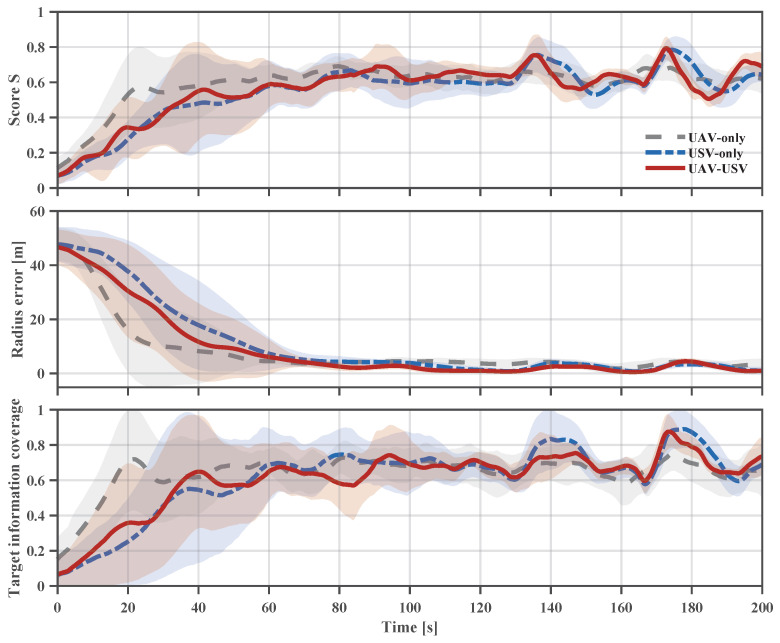
Temporal performance comparison of UAV-only, USV-only, and heterogeneous UAV–USV teams.

Figure 9 compares the average performance of UAV-only, USV-only, and UAV–USV teams in terms of convergence time, post-stabilization score, radius error, information coverage, visual connectivity, control effort, and composite value. The dynamic curves in in Figure 9 show that the UAV-only team converges quickly but with higher control activity, while the USV-only team is smoother but slower. The heterogeneous UAV–USV team achieves the most favorable balance between convergence, radius accuracy, and stable behavior.

**Figure 10:**
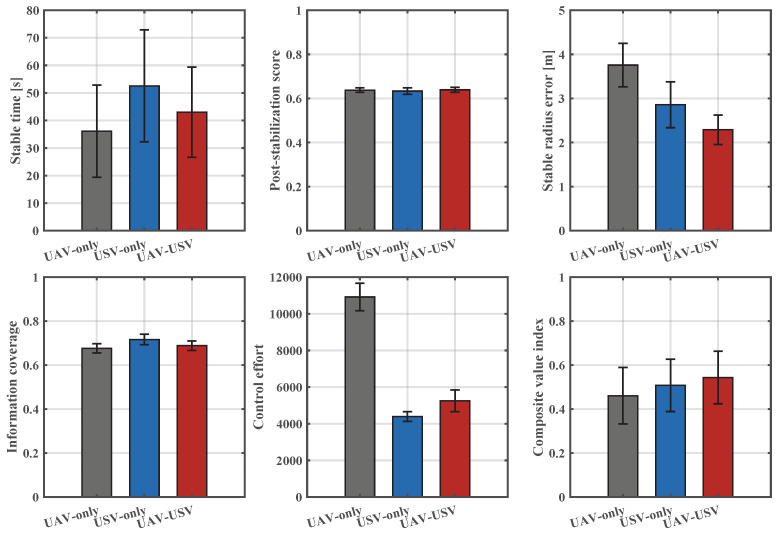
Statistical comparison of homogeneous and heterogeneous platform configurations over multi-seed simulations.

The bar comparison in in Figure 10 confirms that heterogeneity is most beneficial for task-critical encirclement accuracy and composite value rather than for every individual metric. This is consistent with the biological interpretation: mixed functional capabilities improve group-level performance through complementarity.

Table 9 quantifies this effect. UAV–USV obtains the highest post-stabilization score and the lowest stable radius error among the three platform configurations. Although UAV-only reaches stable encirclement faster and USV-only yields lower control effort, the heterogeneous team provides the best overall trade-off.

**Figure 11:**
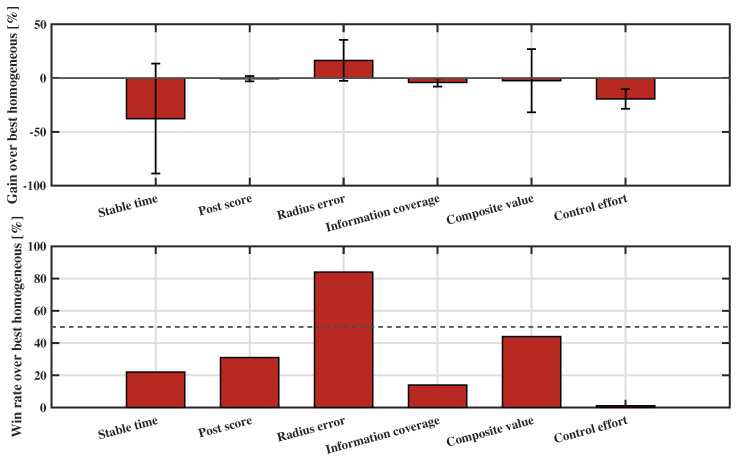
Performance gain of the heterogeneous UAV–USV team over homogeneous baselines.

The gain is calculated relative to the best homogeneous baseline under the same random seed, providing a conservative comparison of heterogeneous complementarity. Figure 11 shows that the UAV–USV team consistently improves the most task-critical metric, radius error, relative to homogeneous baselines. The conservative per-seed best-baseline comparison should therefore be interpreted as a strict test: even under this reference, the heterogeneous system retains a clear advantage in encirclement accuracy.

#### Role Complementarity in the Heterogeneous Team

**Figure 12:**
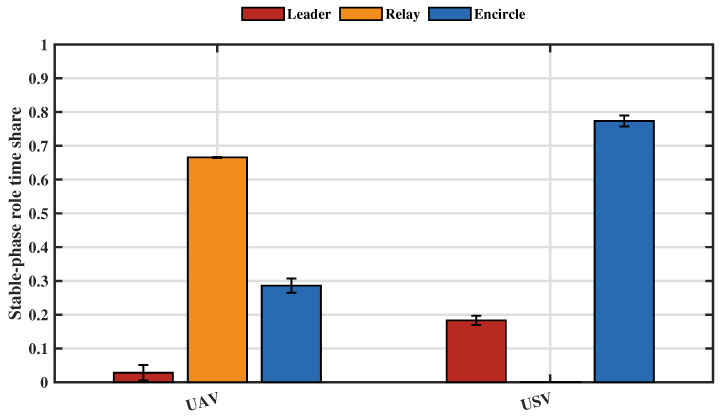
Stable-phase role allocation shares of UAVs and USVs in the heterogeneous team.

Figure 12 reveals a clear emergent division of labor. UAVs spend most of the stable phase as relay agents, using their mobility and observation flexibility to maintain visual information paths. USVs spend most of the stable phase as encircling agents, exploiting their smoother motion to maintain the surrounding ring. This role complementarity explains why the heterogeneous team obtains the lowest radius error and highest composite value: UAVs support information flow and fast adjustment, while USVs provide persistent geometric enclosure. 

Overall, the experiments verify that GB-VCE improves encirclement by coupling biomimetic role growth with visual connectivity preservation. The method achieves better spatial accuracy, more reliable target-information propagation, smoother role differentiation, and stronger heterogeneous complementarity than the evaluated baselines. The organization and definitions of the simulation data supporting the reported statistical results are provided in Appendix A.

## 7. Conclusions and Future Work

This paper proposed GB-VCE, a wolf-pack-inspired distributed encirclement framework for heterogeneous UAV–USV teams operating under visual connectivity constraints. The method translates biological role differentiation into a two-layer robotic coordination architecture: the upper layer accumulates local growth evidence to select leader, relay, searcher, and encircler roles, while the lower layer converts the selected role into bounded motion commands for heterogeneous platforms. By using visual links and relay relations as coordination resources, GB-VCE avoids dependence on centralized fusion or persistent all-to-all communication.

Simulation results over 100 paired-seed runs demonstrate that GB-VCE improves both convergence and stable encirclement quality. Compared with fixed-role and static-leader baselines, the proposed method achieves the highest final success metric, the lowest final radius error, faster stable encirclement, and higher target-information coverage. The heterogeneous verification further shows that UAV–USV cooperation provides a better balance than UAV-only or USV-only teams: UAVs mainly emerge as relay agents that maintain information flow, while USVs mainly serve as encircling agents that stabilize the surrounding geometry. These results confirm that biomimetic role growth can transform platform heterogeneity into measurable cooperative performance gains.

The current study still has several limitations. The validation is simulation-based, and the target dynamics, sensing uncertainty, communication delay, localization error, environmental disturbances, and aerodynamic and hydrodynamic effects are simplified. In addition, the current framework considers planar motion and single-target encirclement. Future work will introduce a high-fidelity air–sea simulation environment, hardware-in-the-loop validation, and physical UAV–USV experiments. Three-dimensional UAV motion, multi-target encirclement, and more realistic sensing, communication, and environmental models will also be investigated.

## Figures and Tables

**Table 1:** Comparison of representative methods for cooperative encirclement and heterogeneous coordination.

Method	Main Idea	Advantages	Limitations
Geometric target enclosing [1,2]	Maintain desired bearing, distance, or angular distribution around the target	Interpretable control structure and direct encirclement objective	Often assumes continuous observation or sufficient state information
Learning-based coordination [3,4,5]	Learn cooperative policies through reinforcement learning or multi-agent value/policy decomposition	Adaptive to complex dynamics and environmental uncertainty	Role assignment is often implicit and difficult to interpret
Vision/connectivity-aware coordination [10,11,12]	Preserve visual links, relay relations, or network connectivity among agents	Improves robustness under constrained communication	Usually focuses on connectivity maintenance rather than encirclement role evolution
Temporal role allocation [13,14]	Introduce continuity or stability into multi-agent role switching	Reduces frequent role oscillation	Not specifically designed for heterogeneous visual encirclement
Biological collective hunting [15]	Use natural role specialization and adaptive group hunting as inspiration	Provides interpretable principles for distributed encirclement	Requires formalization for robotic control
Proposed method [16,17,18]	Growth-based role evolution with visual connectivity preservation	Adaptive, distributed, interpretable, and suitable for UAV–USV encirclement	Requires evaluation of growth state and local visual topology

The references listed in each row denote representative studies or conceptual foundations related to the corresponding methodological category. For the proposed-method row, these references indicate biological inspiration and visual-connectivity-related foundations, rather than previous presentations of the proposed GB-VCE framework. The original contribution of this work lies in integrating growth-based role evolution and visual connectivity preservation into a unified distributed encirclement framework for heterogeneous UAV–USV systems.

**Table 2:** Simulation environment parameters.

Parameter	Value	Description
Environment size	$240\times 240\;m^{2}$	Length × width
$N_{\mathrm{UAV}}$	3	Number of UAVs
$N_{\mathrm{USV}}$	5	Number of USVs
*N*	8	Total number of agents
$x_{T}\left(0\right)$	${\left[40,-30,1.2,0.4\right]}^{⊤}$	Initial target state ${\left[x_{T},y_{T},v_{T}^{x},v_{T}^{y}\right]}^{⊤}$
$v_{\mathrm{UAV}}^{\max}$	3.6 m/s	Maximum linear velocity of UAVs
$v_{\mathrm{USV}}^{\max}$	2.0 m/s	Maximum linear velocity of USVs
$\omega^{\max}$	1.2 rad/s	Maximum angular velocity
$a_{\mathrm{UAV}}^{c,\max}$	$2.2\;m/s^{2}$	Maximum forward acceleration of UAVs
$a_{\mathrm{UAV}}^{c,\min}$	$-2.8\;m/s^{2}$	Maximum braking acceleration of UAVs
$a_{\mathrm{USV}}^{c,\max}$	$0.85\;m/s^{2}$	Maximum forward acceleration of USVs
$a_{\mathrm{USV}}^{c,\min}$	$-1.10\;m/s^{2}$	Maximum braking acceleration of USVs
$\alpha_{\mathrm{UAV}}^{\max}$	$3.8\;\mathrm{rad}/s^{2}$	Maximum angular acceleration of UAVs
$\alpha_{\mathrm{USV}}^{\max}$	$1.10\;\mathrm{rad}/s^{2}$	Maximum angular acceleration of USVs
$R_{\mathrm{UAV}}$	38 m	Visual sensing range of UAVs
$R_{\mathrm{USV}}$	30 m	Visual sensing range of USVs
$\phi_{i}$	85°	Half field-of-view angle
$R_{\mathrm{enc}}$	16 m	Desired encirclement radius
$R_{\mathrm{close}}$	3.5 m	Close-distance threshold for encirclement evaluation
$\Gamma_{\mathrm{th}}$	0.30	Target-information coverage threshold
$S_{\mathrm{th}}$	0.70	Stable encirclement success threshold
$e_{r,\mathrm{th}}$	7.0 m	Stable encirclement radius-error threshold
$K_{\mathrm{hold}}$	12 steps	Required holding steps for stable encirclement

**Table 3:** Key motion-control hyperparameters.

Parameter	Value	Description	Evaluated Range	Rationale
$k_{v}$	1.80	Linear-velocity tracking gain	1.0–2.5	Balance tracking speed and smoothness
$k_{\omega }$	2.40	Angular-velocity tracking gain	1.5–3.0	Reduce heading lag without angular oscillation
$k_{L}$	1.10	Leader target-tracking gain	0.6–1.4	Maintain target guidance and formation flexibility
kRe	0.80	Relay guidance gain	0.4–1.2	Preserve visual relay paths without overconstraint
$k_{\mathrm{align}}$	0.12	Local heading-alignment gain	0.05–0.25	Improve local consistency while avoiding swarm rigidity
$T_{\mathrm{lost}}$	8.0 s	Maximum duration for short-term target prediction	4–12 s	Avoid stale target estimates after long target loss

The selected values were chosen through preliminary paired-seed simulations to balance convergence speed, control smoothness, and visual connectivity preservation.

**Table 4:** Key role-evolution hyperparameters.

Parameter	Value	Description	Evaluated Range	Rationale
$\eta_{g}$	1.10	Growth driving gain	0.7–1.5	Control response speed of role evidence
$\lambda_{g}$	0.30	Natural decay coefficient of growth state	0.1–0.5	Smooth role evolution and suppress transient changes
$w_{O}$	0.42	Weight of target observation quality	0.30–0.55	Emphasize target observation in role growth
$w_{C}$	0.28	Weight of visual connectivity contribution	0.15–0.40	Encourage information propagation and relay contribution
$\Delta_{L}$	0.10	Leadership switching margin	0.05–0.20	Prevent frequent leader switching while retaining adaptivity
$T_{\mathrm{hold}}$	6 steps	Minimum holding steps for leader switching	3–10 steps	Require persistent superiority before leader replacement
NRe	2	Number of relay agents	1–3	Provide relay support without reducing encirclers excessively

The selected values were chosen to balance target observation, visual connectivity preservation, role stability, and heterogeneous role complementarity.

**Table 5:** Computational time of the evaluated simulation studies over 100 independent runs.

Simulation Study	Method/Configuration	Time Per Run (s)	Average Time Per Step (ms)
Strategy comparison	Fixed-Role	1.1371±0.1591	1.136
	Static-Leader	2.0811±0.3374	2.079
	Proposed GB-VCE	1.5198±0.2040	1.518
Heterogeneous-value study	UAV-only	1.9405±0.3426	1.939
	USV-only	1.4383±0.2609	1.437
	UAV–USV	1.3390±0.1754	1.338

Values are reported as mean ± standard deviation over 100 independent runs. Each run represents a 200 s simulated mission with a sampling interval of 0.2 s, corresponding to 1001 control steps. Wall-clock timing includes simulation and control computations but excludes figure generation and file-output operations.

**Table 6:** Statistical comparison of encirclement performance over 100 independent paired-seed runs.

Metric	Fixed-Role	Static-Leader	Proposed GB-VCE
Final success metric ↑	0.271±0.017 [0.267, 0.274]	0.598±0.139 [0.570, 0.625]	0.644±0.060 [0.632, 0.655]
Mean success score in last 20 steps ↑	0.336±0.002 [0.335, 0.336]	0.544±0.028 [0.539, 0.550]	0.634±0.022 [0.629, 0.638]
Post-stabilization mean score ↑	0.336±0.002 [0.335, 0.336]	0.543±0.033 [0.536, 0.549]	0.640±0.009 [0.638, 0.641]
Final radius error (m) ↓	4.831±0.088 [4.814, 4.848]	2.851±1.064 [2.642, 3.059]	1.040±1.034 [0.837, 1.242]
Post-stabilization radius error (m) ↓	4.078±0.067 [4.065, 4.091]	3.044±0.429 [2.960, 3.128]	2.343±0.297 [2.285, 2.402]
Mean radius error in last 20 steps (m) ↓	4.078±0.067 [4.065, 4.091]	2.826±0.741 [2.680, 2.971]	2.142±0.371 [2.069, 2.214]
Stable encirclement time (s) ↓	N.R. (0/100)	68.29±41.15 [60.10, 76.48] (97/100)	40.65±11.25 [38.44, 42.85] (100/100)
Stable reached rate ↑	0.00	0.97	1.00

Values are mean ± standard deviation with 95% confidence interval in brackets. N.R. denotes that stable encirclement was not reached. For stable encirclement time, statistics are computed only over successful runs; parentheses indicate success count out of 100. ↑ indicates that a higher value is better, whereas ↓ indicates that a lower value is better. Bold values identify the best result for each metric according to this direction.

**Table 7:** Statistical comparison of information coverage, visual connectivity, role adaptation, and control smoothness over 100 independent paired-seed runs.

Metric	Fixed-Role	Static-Leader	Proposed GB-VCE
Final coverage ratio ↑	0.625±0.000 [0.625, 0.625]	0.705±0.219 [0.662, 0.748]	0.726±0.066 [0.713, 0.739]
Post-stabilization coverage ↑	0.605±0.005 [0.604, 0.606]	0.616±0.050 [0.607, 0.626]	0.692±0.018 [0.688, 0.695]
Mean visual connectivity ↑	0.020±0.001 [0.020, 0.020]	0.085±0.032 [0.079, 0.092]	0.085±0.018 [0.082, 0.089]
Mean geometric score in last 20 steps ↑	0.511±0.005 [0.510, 0.512]	0.645±0.073 [0.631, 0.660]	0.728±0.038 [0.720, 0.735]
Mean information score in last 20 steps ↑	0.254±0.003 [0.253, 0.254]	0.490±0.044 [0.482, 0.499]	0.716±0.016 [0.713, 0.719]
Mean saturated visual score in last 20 steps ↑	0.168±0.006 [0.167, 0.170]	0.458±0.139 [0.430, 0.485]	0.368±0.056 [0.357, 0.379]
Role switches ↓	52.16±45.53 [43.24, 61.08]	273.96±54.53 [263.27, 284.65]	130.27±27.71 [124.84, 135.70]
Leader switches ↓	0.00±0.00 [0.00, 0.00]	0.00±0.00 [0.00, 0.00]	8.14±2.95 [7.56, 8.72]
Control smoothness ↓	0.691±0.086 [0.674, 0.707]	1.342±0.105 [1.322, 1.363]	1.083±0.085 [1.067, 1.100]

Values are mean ± standard deviation with 95% confidence interval in brackets. ↑ indicates that a higher value is better, whereas ↓ indicates that a lower value is better. The best value for each metric is bold according to its optimization direction. Although fixed-role has fewer role switches and lower smoothness, it never reaches stable encirclement (see Table 6).

**Table 8:** Statistical significance test between GB-VCE and the static-leader baseline.

Metric	GB-VCE	Static-Leader	Improvement	*p*-Value
Final success metric ↑	0.6435	0.5978	+7.65%	$6.81\times 10^{-3}$
Stable encirclement time (s) ↓	40.85	68.29	+40.18%	$1.22\times 10^{-13}$
Final radius error (m) ↓	1.0397	2.8505	+63.52%	$1.45\times 10^{-12}$
Post-stabilization coverage ↑	0.6919	0.6164	+12.24%	$2.84\times 10^{-15}$
Mean information score in last 20 steps ↑	0.7158	0.4903	+45.99%	$3.90\times 10^{-18}$

The Wilcoxon signed-rank test was conducted using paired-seed simulation results. For benefit-type metrics, a positive improvement means that GB-VCE achieves a higher value. For cost-type metrics, including stable encirclement time and radius error, a positive improvement means that GB-VCE achieves a lower value. ↑ denotes a benefit-type metric for which higher values are better, and ↓ denotes a cost-type metric for which lower values are better.

**Table 9:** Multi-seed statistical comparison of different platform configurations.

Metric	UAV-Only	USV-Only	UAV-USV	Best	Direction
Stable encirclement time [s]	36.11±16.76	52.56±20.28	42.98±16.35	UAV-only	Lower
Post-stabilization score	0.6377±0.0104	0.6333±0.0145	0.6390±0.0114	UAV-USV	Higher
Stable radius error [m]	3.757±0.492	2.856±0.519	2.290±0.334	UAV-USV	Lower
Information coverage	0.6764±0.0209	0.7166±0.0236	0.6886±0.0215	USV-only	Higher
Mean visual connectivity	0.1423±0.0422	0.0982±0.0295	0.0847±0.0192	UAV-only	Higher
Control effort	10,920.4±751.5	4394.6±270.7	5253.3±592.8	USV-only	Lower
Composite value index	0.4608±0.1283	0.5080±0.1191	0.5438±0.1197	UAV-USV	Higher
Mean rank	2.17	2.05	1.78	UAV-USV	Lower
Best count	28	28	44	UAV-USV	Higher
Success rate [%]	100	100	100	All	Higher

The values are reported as mean ± standard deviation over 100 independent random seeds. Bold values indicate the best result for each metric. The composite value index is calculated from normalized convergence, encirclement accuracy, information availability, visual connectivity, and control-effort-related metrics.

## Data Availability

The simulation data supporting the findings of this study are described in Appendix A and provided in the accompanying Supplementary Materials. The Supplementary Files include per-seed results for the strategy-comparison and heterogeneous-value studies, statistical-test outputs, metric definitions, heterogeneous-gain and role-complementarity data, and computational-time records. Additional information is available from the corresponding authors upon reasonable request.

## Supplementary Materials

The following supporting information can be downloaded at: https://www.mdpi.com/article/10.3390/biomimetics11070513/s1. Table S1: Strategy Comparison All Seed Metrics. Table S2: Strategy Comparison Significance Tests. Table S3: Strategy Comparison Metric Definitions. Table S4: Heterogeneous Study All Trials. Table S5: Heterogeneous Gain All Seeds. Table S6: Heterogeneous Role Complementarity.

## Dual-Use Research of Concern Statement

Current research is limited to cooperative robots, which is beneficial for swarm intelligence researches and does not pose a threat to public health or national security. Authors acknowledge the dual use potential of the research involving autonomous robot control technologies and confirm that all necessary precautions have been taken to prevent potential misuse. As an ethical responsibility, authors strictly adhere to relevant national and international laws regarding DURC. Authors advocate for responsible deployment, ethical considerations, regulatory compliance, and transparent reporting to mitigate misuse risks and foster beneficial outcomes.

## Appendix A. Simulation Data Supporting the Statistical Analysis

The simulation data were generated from 100 paired random seeds for each evaluated method or platform configuration. Each run represented a 200 s simulated mission with a sampling interval of 0.2 s, corresponding to 1001 simulation steps. Under the same random seed, the compared methods or platform configurations were evaluated using matched initial-condition and target-motion perturbations.

Two groups of simulation data were generated. The strategy-comparison dataset contains the results of the fixed-role, static-leader, and proposed GB-VCE methods. The heterogeneous-value dataset contains the results of the UAV-only, USV-only, and heterogeneous UAV–USV configurations. The recorded variables include encirclement performance, convergence time, radius error, target-information coverage, visual connectivity, role transitions, control effort, heterogeneous gain, and computational time.

The Supplementary Data files and their correspondence to the results reported in the main text are summarized in Table A1.

The complete per-seed data are provided in machine-readable CSV format. The strategy-comparison dataset contains 300 records, corresponding to 100 seeds and three methods. The heterogeneous-value dataset also contains 300 records, corresponding to 100 seeds and three platform configurations. Summary statistics, confidence intervals, significance-test results, and heterogeneous-gain measures reported in the main text were calculated from these per-seed records.

**Table A1:** Supplementary Data files supporting the reported simulation results.

File	Content
S1_StrategyComparison_AllSeedMetrics.csv	Per-seed performance results for the fixed-role, static-leader, and proposed GB-VCE methods over 100 paired random seeds.
S2_StrategyComparison_SignificanceTests.csv	Paired statistical-test results for the principal performance metrics, including sample sizes and *p*-values.
S3_StrategyComparison_MetricDefinitions.csv	Definitions, units, and evaluation directions of the principal metrics used in the strategy-comparison study.
S4_HeterogeneousStudy_AllTrials.csv	Per-seed performance results for the UAV-only, USV-only, and heterogeneous UAV–USV configurations.
S5_HeterogeneousGain_AllSeeds.csv	Per-seed performance gains of the heterogeneous UAV–USV configuration relative to the best homogeneous configuration.
S6_HeterogeneousRoleComplementarity.csv	Per-seed UAV and USV role-time shares for the leader, relay, and encircler roles.

**Table A2:** Principal data fields used in the statistical analyses.

Field	Unit	Description
Seed	–	Random-seed index used to generate the matched simulation condition.
Method/Configuration	–	Evaluated control method or platform configuration.
FinalSuccessMetric	–	Composite encirclement score at the final simulation step.
StableEncirclementTime	s	First time at which the prescribed stable-encirclement conditions were continuously satisfied.
RadiusError	m	Deviation between the achieved and desired encirclement radii.
TargetInformationCoverage	–	Fraction of agents with direct or relayed access to target information.
MeanVisualConnectivity	–	Mean visual connectivity measure of the agent team.
RoleSwitches	–	Total number of role transitions during one simulation run.
LeaderSwitches	–	Total number of leader replacements during one simulation run.
ControlEffort	–	Accumulated motion-control cost over the simulated mission.
CompositeValueIndex	–	Normalized aggregate index combining convergence, accuracy, information availability, visual connectivity, and control effort.
